# Robot-Assisted Arm Training versus Therapist-Mediated Training after Stroke: A Systematic Review and Meta-Analysis

**DOI:** 10.1155/2020/8810867

**Published:** 2020-10-27

**Authors:** Zejian Chen, Chun Wang, Wei Fan, Minghui Gu, Gvzalnur Yasin, Shaohua Xiao, Jie Huang, Xiaolin Huang

**Affiliations:** ^1^Department of Rehabilitation Medicine, Tongji Hospital, Tongji Medical College, Huazhong University of Science and Technology, Wuhan 430030, China; ^2^World Health Organization Cooperative Training and Research Center, Wuhan 430030, China

## Abstract

**Background:**

More than two-thirds of stroke patients have arm motor impairments and function deficits on hospital admission, leading to diminished quality of life and reduced social participation. Robot-assisted training (RAT) is a promising rehabilitation program for upper extremity while its effect is still controversial due to heterogeneity in clinical trials. We performed a systematic review and meta-analysis to compare robot-assisted training (RAT) versus therapist-mediated training (TMT) for arm rehabilitation after stroke.

**Methods:**

We searched the following electronic databases: MEDLINE, EMBASE, Cochrane EBM Reviews, and Physiotherapy Evidence Database (PEDro). Studies of moderate or high methodological quality (PEDro score ≥4) were included and analyzed. We assessed the effects of RAT versus TMT for arm rehabilitation after stroke with testing the noninferiority of RAT. A small effect size of −2 score for mean difference in Fugl-Meyer Assessment of the Upper Extremity (FMA-UE) and Cohen's *d* = −0.2 for standardized mean difference (SMD) were set as noninferiority margin.

**Results:**

Thirty-five trials with 2241 participants met inclusion criteria. The effect size for arm motor impairment, capacity, activities of daily living, and social participation were 0.763 (WMD, 95% CI: 0.404 to 1.123), 0.109 (SMD, 95% CI: −0.066 to 0.284), 0.049 (SMD, 95% CI: −0.055 to 0.17), and −0.061 (SMD, 95% CI: −0.196 to 0.075), respectively.

**Conclusion:**

This systematic review and meta-analysis demonstrated that robot-assisted training was slightly superior in motor impairment recovery and noninferior to therapist-mediated training in improving arm capacity, activities of daily living, and social participation, which supported the use of RAT in clinical practice.

## 1. Introduction

Stroke is a leading cause of mortality and disability worldwide according to The Global Burden of Diseases, Injuries, and Risk Factors Study (GBD) [1–3]. On hospital admission after stroke, more than two-thirds of the patients have upper extremity motor impairments and function deficits. The inability of upper extremity in daily life leads to lower perceived health-related quality of life and reduced social participation [4, 5]. According to the Guidelines for Adult Stroke Rehabilitation and Recovery from American Stroke Association [4], intensive task-specific training and activities of daily living (ADL) training are the most recommended interventions with strong evidence for brain neuroplasticity and arm recovery [6]. Hence, not only impairment-oriented but also function-oriented training is of importance to improve arm motor impairment, capacity, activities of daily living, and social participation in stroke rehabilitation [7].

Robot-assisted training is an innovative exercise-based therapy that enables the implementation of highly repetitive, intensive, adaptive, quantifiable, and task-specific arm training with feedback and motivation for boosting brain neuroplasticity [8–10]. Robotic devices, unlike humans, programmed to perform in different functional modes with a single click can relieve the burden of the shortage of rehabilitation providers and resources without fatigue [11].

For the current state, the effects of robot-assisted training reach no consensus with mixed controls. The 2018 Cochrane review indicated that electromechanical and robot-assisted arm training improved arm muscle strength, arm function, and ADL without increasing dropout rate as well as intervention-related adverse events, compared with various conventional interventions, usual care, or sham intervention [12]. However, another meta-analysis with various controls also found that robot-assisted training showed positive effects in motor control and muscle strength but not in basic activities of daily living [13]. Rodgers conducted a multicenter trial, robot-assisted training for the upper limb after stroke (RATULS), comparing robot-assisted training, enhanced upper limb therapy, and usual care in 770 participants. However, the results did not support the use of robot-assisted training in routine clinical practice because it led to improvement in motor impairment compared with usual care but not in arm function or ADL. Meanwhile, enhanced therapy did lead to improvements in impairment, mobility, and ADL compared with usual care [14]. The Veterans Affairs (VA) *Robotics Trial* demonstrated that robot-assisted training improved much in motor impairment, capacity, and ADL than usual care while it was not statistically different compared with enhanced upper limb therapy [15]. In rehabilitation, usual care is heterogeneous for many reasons: multiple treatments, different order of therapies, and personal behaviors that affect the outcomes [16]. Since the results were inconsistent and confusing, we hypothesized that the actual effects of robot-assisted training might be confound with heterogeneous controls in previous meta-analysis [9, 12, 13, 17–19].

When interpreting effects, robot-assisted training needs to be compared with positive and evidence-based control, especially with arm training mediated by therapists [4, 6, 20, 21], as the rehabilitation robot was designed as a potential alternative tool with the standardized training environment to reduce the burden of therapists [22, 23]. The comparison between robot-assisted training (RAT) and therapist-mediated training (TMT) can be considered as a strict test because TMT (positive control) has been supported by mass evidence [8]. In the manner of practical utilization, upper limb robots were manufactured to provide intensive training conventionally mediated by therapists. If RAT was noninferior to TMT, it might be reasonable to perform RAT in arm stroke rehabilitation according to actual circumstances [24]. Furthermore, previous meta-analyses did not address the effects of robot-assisted training on social participation, which is one of the most pivotal outcomes in rehabilitation after stroke [25].

This systematic review and meta-analysis aimed to compare the clinical effects of robot-assisted training with therapist-mediated training on arm motor impairment, capacity, activities of daily living, and social participation after stroke and test the noninferiority of robot-assisted training compared with therapist-mediated training. Besides, as trial design, training characteristics, and participant characteristics may have influences on the clinical outcomes, we conducted subgroup analyses and meta-regression to examine methodological discrepancies across the included trials.

## 2. Methods

### 2.1. Study Design

The study was reported based on the preferred reporting items for systematic reviews and meta-analyses (PRISMA) statement [26] and registered with PROSPERO (international prospective register of systematic reviews, CRD42019137203). The design and statistical analyses of the study were under the framework of noninferior test [27]. Articles were searched for effect size and minimal clinically important difference (MCID) of the outcome measures to define a prior noninferiority margin for the test [6, 20, 21, 28–30]. As a result, a small effect size of −2 points for mean difference in Fugl-Meyer Assessment of the Upper Extremity (FMA-UE) (MCID: 4 points for acute stroke patients) and Cohen's *d* = −0.2 for standardized mean difference (SMD) were set as noninferiority margin. We included randomized controlled trials (RCTs) and randomized controlled crossover trials (analyzing the first study period as a parallel-group trial). Therapist-mediated training was defined as impairment-oriented or function-oriented upper limb training tailored by therapists to the individual's impairment or requirements, including conventional occupational therapy, physical therapy, task-specific training, ADL training, and constraint-induced movement therapy (CIMT). In addition, robot-assisted training was either the only training program or performed in combination with conventional arm training in a trial setting; hence, we defined the included trials as “alone” or “add-on” design, respectively, to explore its influence on the outcomes by subgroup analysis.

### 2.2. Search Strategy

We searched the following electronic databases: MEDLINE, EMBASE, Cochrane EBM Reviews, and Physiotherapy Evidence Database (PEDro) up to October 2019. Indexing terms and free-text words included “Robot-Assisted Therapy” (robotics, exoskeleton, and end-effector), “upper limb” (upper extremity, arm, hand, shoulder, elbow, forearm, finger, and wrist), “stroke” (cerebrovascular accident), and “randomized controlled trial” (RCT, randomized controlled crossover trials) (see Supplementary Materials for search strategy). The search was limited to English language articles. We screened the reference lists of the included articles and searched published systematic review or meta-analysis for any missed studies.

### 2.3. Inclusion and Exclusion Criteria

The inclusion criteria were (1) randomized controlled trials or randomized controlled crossover trials; (2) patients diagnosed with stroke and having upper limb motor dysfunction; (3) studies investigating the effects of robot-assisted arm rehabilitation compared with therapist-mediated training; (4) outcome measures including arm motor impairment, capacity, and activities of daily living or social participation. The exclusion criteria were (1) RCTs with mixed populations (such as traumatic brain injury and stroke); (2) control groups in the study not containing therapist-mediated training; (3) participant number lesser than 10 in any group; (4) studies receiving a PEDro scale rating of “poor,” defined as 3 or less.

### 2.4. Selection of Studies

Two authors (ZJ Chen and C Wang) read the titles and abstracts independently and eliminated obviously irrelevant studies. Then, they read the full text of the articles to select the remaining articles. Disagreements were resolved by discussion or by consultation with an adjudicator (XL Huang) when necessary. Reference lists of included RCTs, and relevant systematic and narrative reviews, were screened for relevant publications.

### 2.5. Assessment of Methodological Quality and Bias Risk

Pairs of reviewers (ZJ Chen and C Wang; W Fan and MH Gu) used the PEDro scoring system to assess the methodological quality and risk of bias of the included articles when the score was not available from the PEDro database [31]. The PEDro scale includes 10 items for assessment of trial quality based on whether the trials report the randomization procedure, concealed allocation, blinding of patients, blinding of assessors, adequate follow-up, intention-to-treat analysis, between-group comparability, between-group statistical comparison, and point estimate and variability [32, 33]. PEDro scores of four points or more were classified as “sufficient quality,” whereas studies with three points or less were classified as “insufficient quality” and were excluded from the meta-analysis subsequently.

### 2.6. Data Extraction

Pairs of reviewers (ZJ Chen and C Wang; W Fan and MH Gu) independently extracted data from the included studies and resolved disagreement about data extraction by consensus. Data extracted included methodological quality, participants' characteristics, trial design setting, training characteristics, robot characteristics, and outcome measures. We contacted authors of relevant publications for data when means and/or SDs change were not accessible.

### 2.7. Outcome Measures

- *Primary outcomes.* The primary outcome of the study was motor impairment; we used Fugl-Meyer Assessment of the Upper Extremity (FMA-UE) as measurement [34].
- *Secondary outcomes.* The secondary outcomes of the study were upper limb capacity, activities of daily living, and social participation [35, 36]. As is defined in International Classification of Functioning, Disability and Health (ICF) framework, capacity describes “what a person does in a situation in which the effect of the context is absent or made irrelevant, such as in a standardized evaluation setting” [36]. Due to numerous outcome measures used across trials, the review authors implemented the selection process of measure for quantitative pooling. If more than one measure was available, we prioritized measures as follows: Action Research Arm Test (ARAT), Wolf Motor Function Test (WMFT), Box and Blocks Test (BBT), Nine Hole Peg Test (9-HPT), Chedoke Arm and Hand Activity Inventory (CAHAI), Arm Motor Ability Test (AMAT), and any other available scales.

We assessed activities of daily living by prioritizing Functional Independence Measure (FIM), Barthel Index (BI), Motor Activity Log (MAL), and modified Rankin Scale (mRS). We assessed social participation with the order of priority by Stroke Impact Scale (SIS) and Medical Outcomes Study Short Form 36 (SF-36).

### 2.8. Statistical Analysis

STATA 14.0 was used in this systematic review and meta-analysis. The mean and standard deviation (SD) of the change scores for each outcome measure between the baseline and posttreatment were extracted in the experimental and control groups. If different outcome scales were available, we combined the results using standardized mean difference (SMD) and 95% confidence intervals (CIs). Otherwise, we used the mean difference (MD) with 95% CI for comparison with the minimal clinically important difference (MCID).

We performed the heterogeneity test using the *I*^2^ statistic. According to related articles, an *I*^2^ greater than 50% represented substantial heterogeneity, and the random-effect models were used for data analysis [37]. Otherwise, we performed the fixed effect model. Using Cohen's criteria, effect sizes were interpreted as small (≤0.2), moderate (0.2–0.8), or large (≥0.8). Data were analyzed using an intent-to-treat framework; therefore, sample sizes at the baseline instead of postintervention were considered in the calculation of effect size.

When heterogeneity appeared, we evaluated whether treatment effects for the outcomes were robust in subgroup analyses and metaregression [38]. We conducted subgroup analyses in the following subgroups: trial design setting (add-on design or alone-design) and training characteristics (robot type, training part, and training side) with univariable metaregression models testing of interaction between treatment effect and these characteristics. Forest plot were employed as a means of graphically representing a meta-analysis of the overall results of the included trials. Besides, metaregression was performed for participant characteristics (time after stroke, age), training time, and publication year. We inspected funnel plots and used Egger regression test for all outcomes in order to assess the risk of publication bias. *P* < 0.05 was considered to indicate statistical significance.

## 3. Results

**Figure 1:**
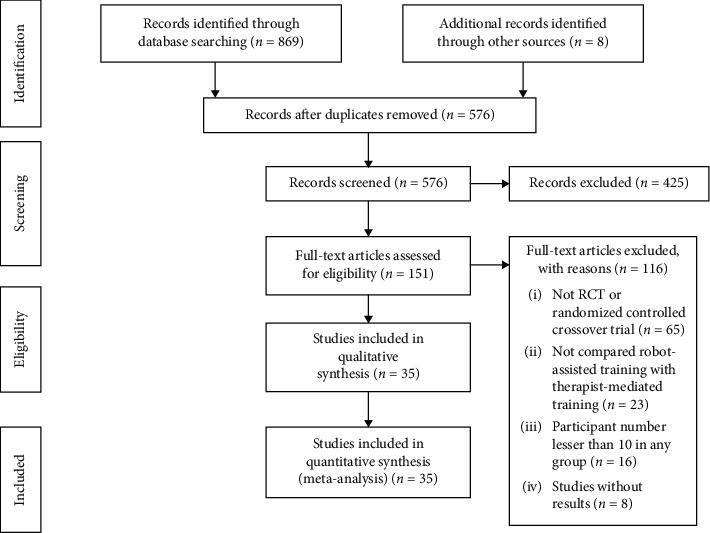
PRISMA flow diagram.

Thirty-five trials with 2241 participants were identified as meeting our inclusion criteria and suitable for quantitative analysis (Figure 1). The studies varied in size, trial design, training characteristics, and participant characteristics. Study sizes ranged from 20 to 770. Twenty-five trials were add-on design and ten were alone-design. Time per session ranged from 30 minutes to 5 hours. Duration of the intervention ranged from 2 weeks to 12 weeks. We multiplied training time per week by duration to get the total training time, ranging from 4.5 hours to 300 hours. The quality of the included studies ranged from four to eight. Other characteristics were documented in the Supplementary Material.

### 3.1. Upper Limb Motor Impairment

**Figure 2:**
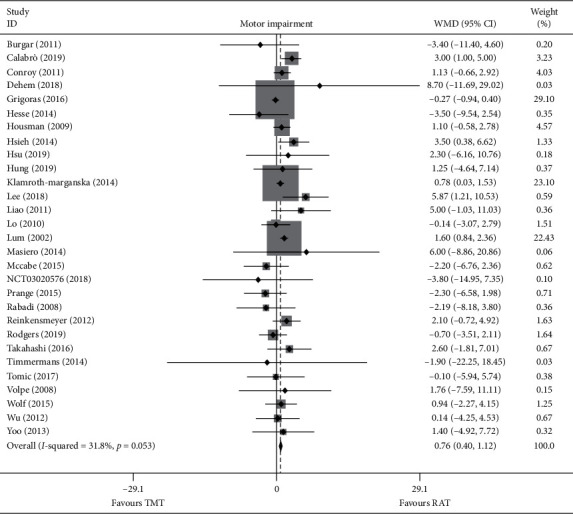
Forest plot of RAT vs. TMT on motor impairment. The each grey area is proportional to the study's weight in the meta-analysis. The overall effect is represented on the plot as a dashed vertical line and the downmost diamond.

**Figure 3:**
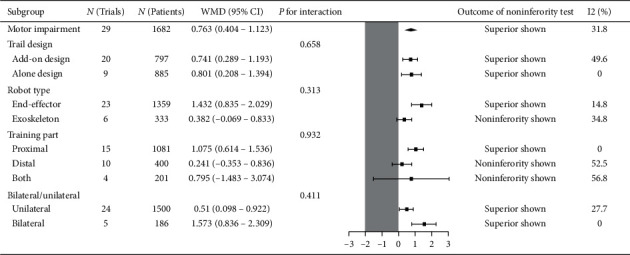
Subgroup results of RAT vs. TMT on motor impairment.

Twenty-nine trials recruiting 1682 participants measured upper limb motor impairment (Figure 2). Robot-assisted training showed a statistically significant mean effect size in motor impairment and was superior compared with therapist-mediated training. The pooled WMD (fixed-effects model) for motor impairment was 0.763 (95% CI: 0.404 to 1.123, *P* < 0.001, level of heterogeneity *I*^2^ = 31.8%). The treatment effect for add-on design trials (20 trials, 797 participants) was significant and superior: WMD = 0.741, 95% CI: 0.289 to 1.193, *P*=0.001, *I*^2^ = 49.6%. For the alone-design trials (9 trials, 885 participants), pooling resulted in a significant and superior treatment effect: WMD = 0.801, 95% CI: 0.208 to 1.394, *P*=0.008, *I*^2^ = 0%. However, the tests for subgroup differences (in trial design, robot type, training part, and training side) revealed no significant difference (*P* for interaction: 0.658, 0.313, 0.932 and 0.411, resp.) (Figure 3).

### 3.2. Upper Limb Capacity

**Figure 4:**
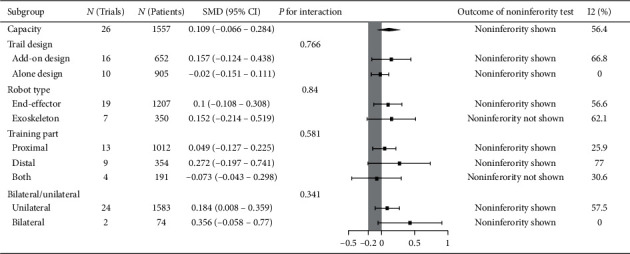
Subgroup results of RAT vs. TMT on capacity.

Twenty-six trials recruiting 1557 participants measured upper limb capacity. Robot-assisted training was not associated with statistically significant improvement in upper limb capacity, but it was noninferior compared with therapist-mediated training. The pooled SMD (random-effects model) for capacity was 0.109 (95% CI: −0.066 to 0.284, *P*=0.02, level of heterogeneity *I*^2^ = 54.6%). The treatment effect for add-on design trials (16 trials, 652 participants) was not significant, but it was noninferior: SMD = 0.157, 95% CI: −0.124 to 0.438, *P*=0.309, *I*^2^ = 66.8%. For the alone-design trials (10 trials, 905 participants), pooling resulted in a not significant but noninferior treatment effect: SMD = −0.02, 95% CI: −0.151 to 0.111, *P*=0.766, I^2^ = 0%. However, the tests for subgroup differences (in trial design, robot type, training part, and training side) revealed no significant difference (*P* for interaction: 0.766, 0.84, 0.581, and 0.341, resp.) (Figure 4).

### 3.3. Activities of Daily Living (ADL)

**Figure 5:**
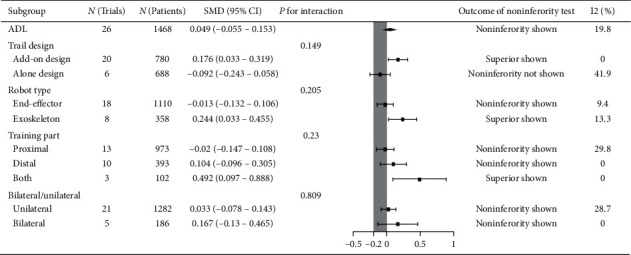
Subgroup results of RAT vs. TMT on ADL.

Twenty-six trials recruiting 1468 participants measured activities of daily living (ADL). Robot-assisted training was not associated with statistically significant improvement in ADL, but it was noninferior compared with therapist-mediated training. The pooled SMD (fixed-effects model) for ADL was 0.0049 (95% CI: −0.055 to 0.17, *P*=0.153, level of heterogeneity *I*^2^ = 19.8). The treatment effect for add-on design trials (20 trials, 780 participants) was significant and superior: SMD = 0.176, 95% CI: 0.033 to 0.319, *P*=0.016, *I*^2^ = 0%. For the alone-design trials (6 trials, 688 participants), pooling resulted in a not significant treatment effect with noninferiority not shown: SMD = −0.092, 95% CI: −0.243 to 0.058, *P*=0.229, *I*^2^ = 41.8%. However, the tests for subgroup differences (in trial design, robot type, training part, and training side) revealed no significant difference (*P* for interaction: 0.149, 0.205, 0.23 and 0.809, resp.) (Figure 5).

### 3.4. Social Participation

**Figure 6:**
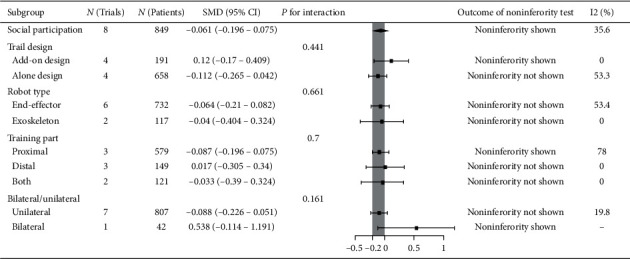
Subgroup results of RAT vs. TMT on social participation.

Eight trials recruiting 849 participants measured social participation. Robot-assisted training was not associated with statistically significant improvement in social participation, but it was noninferior compared with therapist-mediated training. The pooled SMD (fixed-effects model) for social participation was −0.061 (95% CI: −0.196 to 0.075, *P*=0.378, level of heterogeneity *I*^2^ = 35.6%). The treatment effect for add-on design trials (4 trials, 191 participants) was not significant but it was noninferior: SMD = 0.12, 95% CI: −0.17 to 0.409, *P*=0.417, *I*^2^ = 0%. For the alone-design trials (4 trials, 658 participants), pooling resulted in a not significant treatment effect with noninferiority not shown: SMD = −0.112, 95% CI: −0.265 to 0.042, *P*=0.153, *I*^2^ = 53.3%. However, the tests for subgroup differences (in trial design, robot type, training part, and training side) revealed no significant difference (*P* for interaction: 0.441, 0.661, 0.7, and 0.161, resp.) (Figure 6).

### 3.5. Additional Analyses

In metaregression analysis, there was no evidence of effect modification owing to participant characteristics (time after stroke, age) and training time and publication year in the robot-assisted training versus therapist-mediated training for the outcome measures (see Supplementary Material). There was no evidence of publication bias for the assessed outcomes as per the visual or statistical methods (see Supplementary Material).

## 4. Discussion

Prior meta-analysis with mixed controls found positive effects of robot-assisted training in upper motor impairment while inconsistent results in capacity and ADL [9, 12, 13]. However, the sham, blank intervention, or usual care in the control group may confound the pooling results and create bias in favor of robot-assisted training. From a clinical and pragmatic perspective, the purpose of this work was to disentangle the effects of robot-assisted training (RAT) versus therapist-mediated training (TMT) with the noninferiority test.

This systematic review included 35 trials with 2241 participants to evaluate the effect of RAT compared with TMT for upper limb after stroke. Meta-analyses showed that RAT was slightly superior in motor impairment recovery and noninferior to TMT in arm capacity, ADL, and social participation improvement, which supported the utilization of RAT in clinical practice. The tests for subgroup differences (in trial design, robot type, training part, and training side) revealed no significant difference in the outcomes. These findings create a brand new perspective to summarize the effect of RAT in the practical manner. Metaregression of participant characteristics (time after stroke, age), training time, and publication year did not show effect modification. Besides, no evidence of publication bias for the assessed outcomes appeared as per the visual or statistical methods.

Recovery of upper limb depends on neurological recovery, adaptation, and learning new strategies as well as motor programs [7, 39, 40]. Robot-assisted training applies relevant concepts for neuroplasticity and leads to benefits in motor function improvement after stroke [12]. Intensity and control strategies (i.e., active control, passive control, or assist-as-needed control) are both key ingredients in an effective poststroke motor rehabilitation. Although the review fails to compare various amounts of repetitions or control strategies, our study provides the framework for future studies to explore concrete and effective paradigm for RAT. We applied metaregression on total training time and found no modifying effects on the results. Actually, the training intensity means the amount of motor task in a given period [41] so the optimal training parameter (time, sessions, week, and repetitions) with the degree of active participation should be taken into account when interpreting results and need further research.

Robot-assisted training provides standardized task-specific upper extremity exercises but the training effects may differ in various designs of robots. Hence, we conducted novel subgroup analyses in training characteristics (including robot type, training segment, and training side) and found no modifying effects. The results in training segment were in general agreement with the 2018 Cochrane review [12], while it showed inconsistent results in robot type and training side with previous opinions [4, 13]. Different types of robotic device provide various control strategies and training modalities, which may not be tested through observational analysis [42]. Therefore, it is necessary to perform network meta-analysis with head-to-head comparisons or high-quality RCT to detect the differences in the subgroups in the future work.

Meanwhile, since the upper extremity cointervention may augment heterogeneity to the results, we conducted subgroup analysis of add-on design (with cointervention) and alone-design (without cointervention). In the add-on design trials, participants received additional functional training and had the opportunity to transfer the learned motor skills to functional activity in daily routines [43], compared with mere simulated activity in alone-design trials. The results, however, were not statistically significant between the add-on design and alone-design trials. This evidence seemed in contrast with our opinion that generalization of motor improvement to real-world function was important in clinical training. Conroy et al. [44] replaced part of robotic training with therapist-assisted task training, and the results were in line with our perspective that addition of transition-to-task practice placed benefit in hand use and motor task performance. Actually, we found that, in some included alone-design trials, the researchers coupled RAT with real-world objects in task-oriented training [45, 46], which may increase the effects of alone-design trials in upper limb function. Therefore, it may be better to regard robotic device as a training platform consisted of various therapeutic techniques and principles to strengthen the effects, not a tool alone.

The outcomes in the present meta-analysis are based on *the International Classification of Functioning, Disability and Health* (ICF) framework. We selected upper limb motor impairment (body functions and structures level) as primary outcome and ADL (activities level), social participation (participation level), and motor capacity (activities level) as secondary outcomes. We found RAT was slightly superior to TMT in motor impairment (body functions and structures level) and noninferior in the activities level and participation level outcomes. The previous reviews rarely considered the recovery of upper limb capacity and social participation [9, 12, 13]. Upper limb rehabilitation after stroke, in clinical practice, stressed not only motor recovery, but also its generalization and translation into functional tasks in a specific environment, like home, community, company, and so on [47]. Activities of daily and social participation are the pivotal outcomes and mediums of a successful recovery [48]. Participation is defined as the endpoints in the context of recovering from stroke and is known to correlate with domains related to quality of life. However, there is little consensus about the effects of rehabilitation services for stroke survivors once leaving the hospital and living in the community. Our finding suggests a similar effect of RAT and TMT in social participation. Networking robots especially build bridge for group therapy and have benefits in enabling social support, increasing confidence, improving mood, and motivation [49]. Under the circumstances of shortage in therapists and caregivers, RAT, an innovative training platform, improves upper limb motor impairment and translates the learned motor skills into capacity, ADL, and social participation and thus could be considered as a routine therapy in upper limb rehabilitation after stroke.

Some limitations exist in the present study. First, we did not include the articles in languages other than English. Second, the included studies were time-matched instead of repetition-matched because there was no well description about the exact intensity parameters with the degree of active participation in most studies. Third, we did not discuss the safety and cost-effectiveness problems in the article for reasons: on one hand, RAT has been demonstrated safe with high quality of evidence. On the other hand, the cost-effectiveness problems involve the level of economic development of different countries and areas, the offset and regulations of healthcare system, and the development of robotic devices, which are still controversial and deserve further plentiful researches. Finally, apart from training intensity and control strategy, the effects of other ingredients of RAT (i.e., feedback, training contents, and games) may be overlooked in the articles. Future studies may help find out the active ingredients of robot-assisted training, the optimal training paradigm, and the combined protocols in clinical use.

## 5. Conclusions

This systematic review and meta-analysis showed that robot-assisted training was slightly superior in motor impairment recovery (not greater than minimal clinically important difference) and noninferior to therapist-mediated training in improving upper limb capacity, activities of daily living, and social participation, which supports the use of RAT in clinical practice.

## Data Availability

The pooling data used to support the findings of this study are included within the supplementary information file (s).

## Abbreviations

ADL: Activities of daily living
AMAT: Arm motor ability test
ARAT: Action research arm test
BBT: Box and blocks test
BI: Barthel index
CAHAI: Chedoke Arm and Hand Activity Inventory
CIMT: Constraint-induced movement therapy
FIM: Functional independence measure
FMA-UE: Fugl-Meyer Assessment of the Upper Extremity
ICF: International Classification of Functioning, Disability and Health
MAL: Motor activity log
MCID: Minimal clinically important difference
mRS: Modified Rankin Scale
9-HPT: Nine Hole Peg Test
PEDro: Physiotherapy evidence database
PROSPERO: International Prospective Register of Systematic Reviews
RAT: Robot-assisted training
RATULS: Robot-assisted training for the upper limb after stroke
RCT: Randomized controlled trial
SD: Standard deviation
SMD: Standardized mean difference
SF-36: Medical outcomes study short form 36
SIS: Stroke impact scale
TMT: Therapist-mediated training
VA: Veterans Affairs
WMFT: Wolf motor function test.

## Supplementary Materials

Supplementary Materials Appendix 1: preferred reporting items for systematic reviews and meta-analyses (PRISMA) statement. Appendix 2: this file describes the following. I: Forest plots for the outcome measures. II: Metaregression of participant characteristics, training time, and publication year for the outcome measures. III: Funnel plot of standard error for the outcome measures. IV: Supplementary Table 1: PubMed search strategy. V: Supplementary Table 2: Included study characteristics. VI: References of the included studies.
