# Supplementary material for: Robot-Assisted Arm Training versus Therapist-Mediated Training after Stroke: A Systematic Review and Meta-Analysis
Source: J Healthc Eng. 2020 Oct 27;2020:8810867. doi: 10.1155/2020/8810867 (PMC7641296; doi:10.1155/2020/8810867)
Supplement: Supplementary Materials — Appendix 1: preferred reporting items for systematic reviews and meta-analyses (PRISMA) statement. Appendix 2: this file describes the following. I: Forest plots for the outcome measures. II: Metaregression of participant characteristics, training time, and publication year for the outcome measures. III: Funnel plot of standard error for the outcome measures. IV: Supplementary Table 1: PubMed search strategy. V: Supplementary Table 2: Included study characteristics. VI: References of the included studies. [file 8810867.f1.zip › 8810867.f1/SUPPLEMENTAL MATERIAL (2).docx]

**SUPPLEMENTAL MATERIAL**

Robot-assisted arm training versus therapist-mediated training after stroke

A systematic review and meta-analysis

Zejian Chen, Chun Wang, Wei Fan, Minghui Gu, Gvzalnur Yasin, Shaohua Xiao, Jie Huang and Xiaolin Huang.

Department of Rehabilitation Medicine, Tongji Hospital, Tongji Medical College, Huazhong University of Science and Technology; World Health Organization Cooperative Training and Research Center, Wuhan 430030, China.

Correspondence should be addressed to Xiaolin Huang; xiaolinh2006@126.com.

**Ⅰ.Forest plots for the outcome measures.**

**Ⅱ.Meta-regression of participant characteristics, training time and publication year for the outcome measures.**

**Ⅲ.Funnel Plot of Standard Error** **for the outcome measures.**

**Ⅳ.Supplementary Table 1: Pubmed Search Strategy.**

**Ⅴ.Supplementary Table 2: Included study characteristics**

**Ⅵ.References of the included studies**

**Authors Contributions**

ZJ Chen: study concept and design, acquisition of data, statistical analysis, interpretation of data, manuscript writing; C Wang, W Fan, MH Gu, GY: acquisition and analysis of data; SH Xiao, J Huang: critical revision of manuscript; XL Huang: study concept and design, study supervision, critical revision of manuscript.

**Ⅰ.****Forest plots for the outcome measures**

1. Forest plot of motor impairment


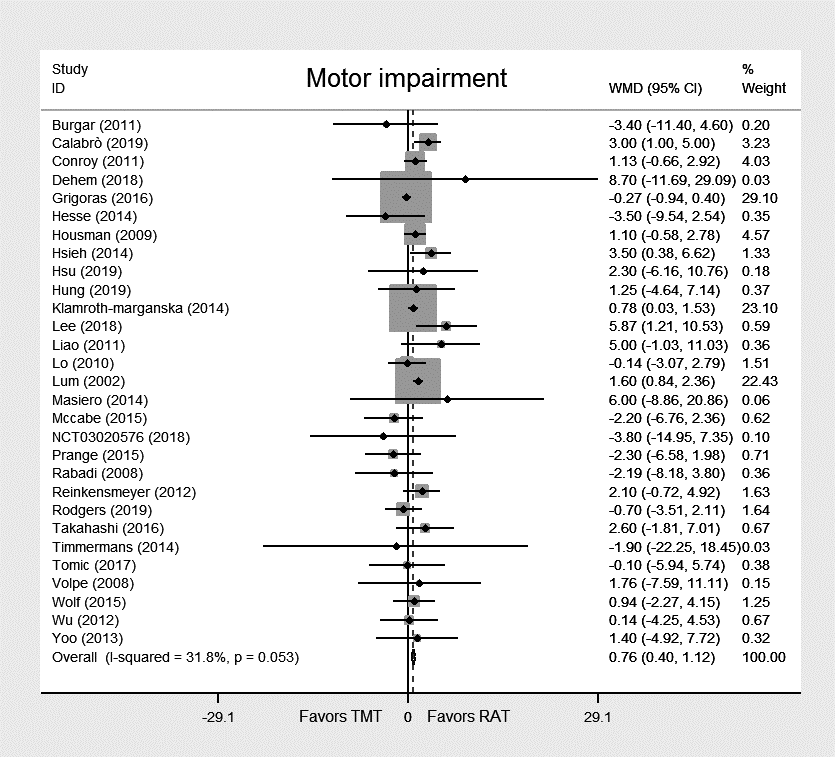


1. Forest plot of capacity


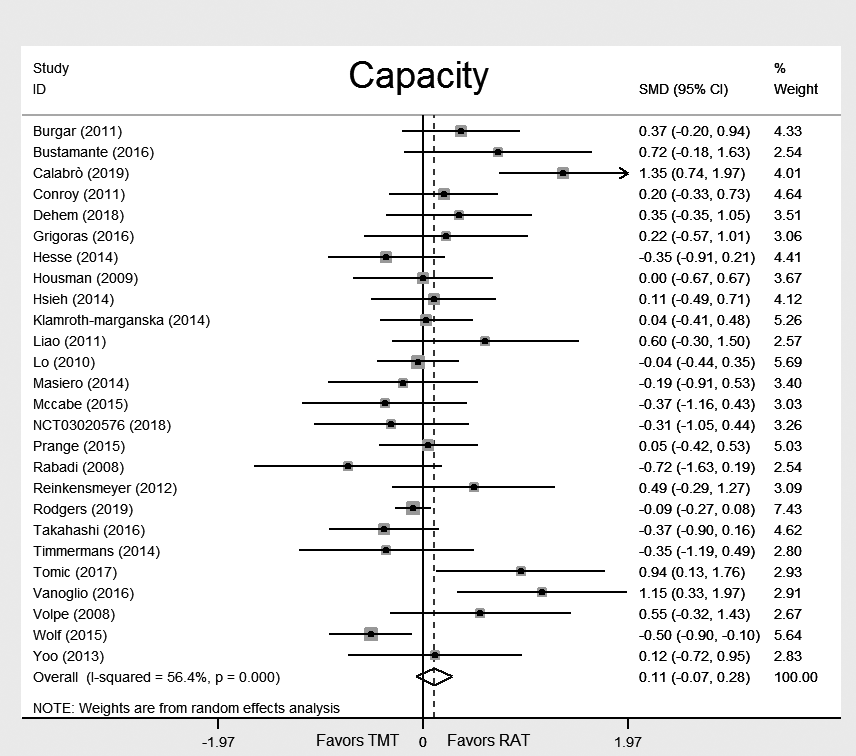


1. Forest plot of activity of daily living (ADL)


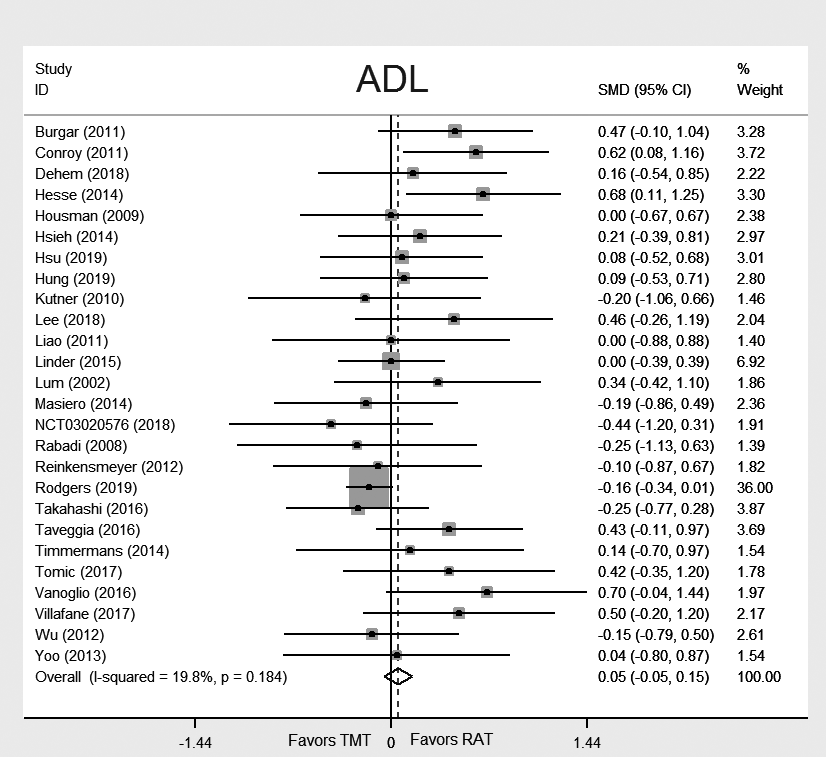


1. Forest plot of social participation


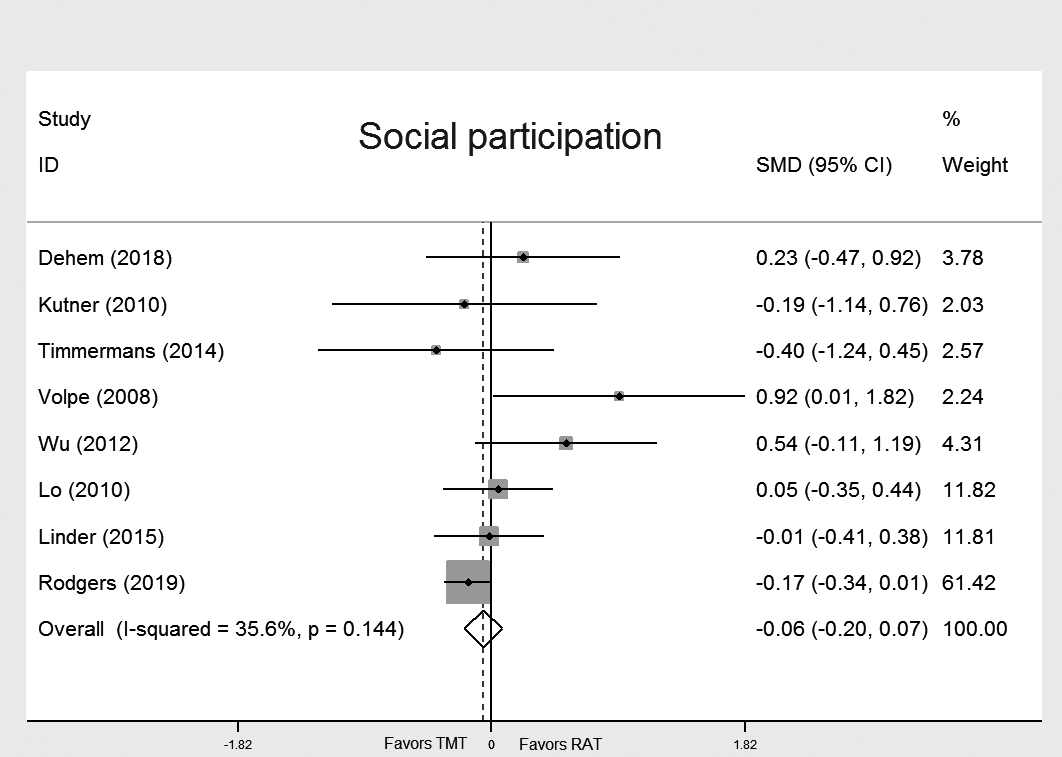


### Ⅱ. Meta-regression of participant characteristics (time post stroke, age), training time and publication year for the outcome measures

1. Meta-regression for motor impairment

2. Meta-regression for capacity

3. Meta-regression for ADL

4. Meta-regression for social participation

**Ⅲ. Funnel Plot of Standard Error for the outcome measures**

1. Funnel Plot and Egger’s test for motor impairment

2. Funnel Plot and Egger’s test for capacity

3. Funnel Plot and Egger’s test for ADL & post-hoc Begg’s test

4. Funnel Plot and Egger’s test for social participation

## Ⅳ.Supplementary Table 1: Pubmed Search Strategy

|  | ("Stroke"[Mesh] OR ("stroke"[MeSH Terms] OR "stroke"[All Fields] OR ("cerebrovascular"[All Fields] AND "accident"[All Fields]) OR "cerebrovascular accident"[All Fields]) OR ("stroke"[MeSH Terms] OR "stroke"[All Fields]) OR (chronic[All Fields] AND ("stroke"[MeSH Terms] OR "stroke"[All Fields]))) |
| --- | --- |
| AND | ((“Robotics”[Mesh] OR Robotics *[tiab] OR robot-aided*[tiab] OR “robot-assisted”[tiab]) ) OR (“computer-assisted”[tiab]) OR “Orthotic devices” [Mesh] OR "Orthotic Devices/methods"[Mesh] OR "Orthotic Devices/therapeutic use"[Mesh] OR "Orthotic Devices/therapy"[Mesh]) OR (Automation[Mesh] OR Robotics/methods*[Mesh] OR Robotics/instrumentation[Mesh] OR Electromechan*[tiab] OR Electro-mechan*[tiab] OR (Robot* AND assis*)[tiab]) OR (Exoskeleton OR End-effector) |
| AND | ("Randomized Controlled Trial"[Publication Type] OR ("randomized controlled trial"[Publication Type] OR "randomized controlled trials as topic"[MeSH Terms] OR "randomized controlled trial"[All Fields]) OR RCT[All Fields] OR ("randomized controlled trial"[Publication Type] OR "randomized controlled trials as topic"[MeSH Terms] OR OR "randomized controlled crossover trial"[All Fields])) |
| AND | (Upper extremity [Mesh] OR Arm [Mesh] OR Elbow [Mesh] OR Forearm [Mesh] OR Hand [Mesh] OR Wrist [Mesh] OR Shoulder [Mesh] OR Finger [Mesh]OR Upper limb[tiab] OR Upper extremity [tiab] OR Arm [tiab] OR Elbow [tiab] OR Forearm [tiab] OR Hand [tiab] OR Wrist [tiab] OR Shoulder [tiab] OR Finger [tiab]) |
| AND | English[lang] |

## Ⅴ. Supplementary Table 2: Included study characteristics

| **Study** | **PEDro scale** | **Stroke type**  **(ischemic/hemorrhage)** | **Time postroke (E/C)** | **Mean age**  **(E/C)** | **Trial**  **Design** | **Robotic**  **Device** | **Type of training(E)** | | **Intensity(E)** | | **Intensity(C)** | | **Duration**  **(week)** | **Outcome Measures** |
| --- | --- | --- | --- | --- | --- | --- | --- | --- | --- | --- | --- | --- | --- | --- |
|  |  |  |  |  |  |  | **Proximal/Distal** | **Bilaterally/**  **Unilaterally** | **Time(min) per session** | **Sessions per week** | **Time(min) per session** | **Sessions per week** |  |  |
| Burgar, 2011.[^1^](#_ENREF_1) | 6 | Both | 17.3/16.6  /10.6d | 62.5/58.6/68.1 | add-on | MIME  (end-effector) | Proximal | Bilaterally | 60 | 3 | 60 | 3 | 8w | FMA, FIM, BI |
| Bustamante, 2016.[^2^](#_ENREF_2) | 4 | Both | ≥6mo | 44.1/64.1 | alone | Robot Gym  (end-effector) | Both | Unilaterally | 120 | 4 | 120 | 4 | 6w | FMA, RLAFT-UE, BBT |
| Calabrò, 2019.[^3^](#_ENREF_3) | 8 | ischemic | 10/10mo | 65/64 | add-on | Amadeo  (end-effector) | Distal | Unilaterally | 45 | 5 | 45 | 5 | 8w | FMA, 9-HPT, EEG, MEP |
| Conroy, 2011.[^4^](#_ENREF_4) | 6 | 51/6 | 3/5/4y | 57/60/56 | alone | InMotion 2.0  (exoskeleton) | Proximal | Unilaterally | 60 | 3 | 60 | 3 | 6w | FMA, WMFT, SIS, Robot metrics |
| Dehem, 2018.[^5^](#_ENREF_5) | 6 | 35/10 | 28.1/27.5d | 67.3/68.6 | add-on | REAplan  (end-effector) | Distal | Unilaterally | 45 | 4 | 45 | 4 | 9w | FMA, BBT, WMFT, ABILHAND, SIS |
| Grigoras, 2016.[^6^](#_ENREF_6) | 6 | 23/2 | 3.69/3.76mo | 62.76/64.75 | add-on | Hybrid system  (exoskeleton) | Distal | Unilaterally | 30 | 12 in total | 30 | 10 in total | 2w | FMA, BBT, SIS |
| Hesse, 2014[^7^](#_ENREF_7). | 7 | 41/9 | 4.5/4.5 w | 71.4/69.7 | add-on | Bi-Manu Track  (end-effector) | Both | Unilaterally | 30 | 5 | 30 | 5 | 4w | FMA, ARAT, BBT, MRC, MAS, BI |
| Housman, 2009.[^8^](#_ENREF_8) | 5 | 17/9/2(Unknown) | 84.5/112.4 mo | 54.2/56.4 | alone | T-WREX  (exoskeleton) | Proximal | Unilaterally | 60 | 3 | 60 | 3 | 8-9w | FMA, MAL, grip strength, RLAFT-UE, ROM |
| Hsieh， 2014.[^9^](#_ENREF_9) | 8 | 27/21 | 20.56/23.56  /27.81mo | 54.41/52.34/54.12 | add-on | Bi-Manu-Track  (end-effector) | Distal | Unilaterally | 90 - 105 | 5 | 90 - 105 | 5 | 4w | FMA, WMFT, MAL, accelerometer |
| Hsu, 2019.[^10^](#_ENREF_10) | 8 | Both | 13.7/14.7mo | 53.1/52.6 | add-on | Bi-Manu-Track  (end-effector) | Distal | Bilaterally | 40 | 3 | 40 | 3 | 4w | MAL, sEMG, FMA |
| Hung, 2019.[^11^](#_ENREF_11) | 8 | 20/10 | 26/20.5  /25mo | 57.5/52.4/55.54 | add-on | Bi-Manu-Track, InMotion 3.0  (end-effector) | Distal | Unilaterally | 90–100 | 5 | 90 - 105 | 5 | 4w | FMA, MAS, MRC, MAL |
| Klamroth, 2014.[^12^](#_ENREF_12) | 8 | Both | 52/40 mo | 55/58 | alone | ARMin  (exoskeleton) | Proximal | Unilaterally | 45 | 3 | 45 | 3 | 8w | FMA, WMFT, MAS, MAL, SIS |
| Kutner, 2010.[^13^](#_ENREF_13) | 6 | 12/5 | 269.6/184.1d | 61.9/51.0 | add-on | Hand Mentor  (exoskeleton) | Distal | Unilaterally | 30h in total | | 30h in total | | 3w | SIS |
| Lee, 2018.[^14^](#_ENREF_14) | 6 | 17/13 | 5/5mo | 52.07/50.27 | add-on | REJOYCE robot  (end-effector) | Both | Unilaterally | 30 | 5 | 30 | 5 | 8w | FMA, MBI |
| Liao, 2011.[^15^](#_ENREF_15) | 8 | Both | 22.9/22.2 mo | 55.51/54.56 | add-on | Bi-Manu-Track  (end-effector) | Distal | Bilaterally | 90 - 105 | 5 | 90 - 105 | 5 | 4w | FMA, FIM, Arm activity ratio, MAL, ABILHAND |
| Linder, 2015.[^16^](#_ENREF_16) | 6 | Both | 117.0/125.6d | 59.4/55.5 | add-on | Hand Mentor Pro  (exoskeleton) | Distal | Unilaterally | 120 | 5 | 120 | 5 | 8w | SIS |
| Lo, 2010.[^17^](#_ENREF_17) | 8 | 108/19 | 3.6/4.8/6.2y | 66/64/63 | alone | MIT-MANUS  (end-effector) | Both | Unilaterally | 60 | 3 | 60 | 3 | 12w | FMA, WMFT, MAS, SIS, pain |
| Lum, 2002.[^18^](#_ENREF_18) | 6 | Both | 30.2/28.8mo | 63.2/65.9 | add-on | MIME  (end-effector) | Proximal | Bilaterally | 50 | 3 | 50 | 3 | 8w | FMA, FIM, BI, reach extend, strength |
| Masiero, 2014.[^19^](#_ENREF_19) | 6 | 26/6 | 8.34/10.23d | 65.60/66.83 | add-on | NeReBot  (end –effector) | Proximal | Unilaterally | 40 | 5 | 40 | 5 | 5w | MRC, FMA, FIM, BBT, FAT, MAS |
| Mccabe, 2015.[^20^](#_ENREF_20) | 6 | Both | 1-3y:n=9/8/10;  ≥4y:n=3/3/2 | 21-49y:n=2/2/3;50-81y:n=10/9/9 | add-on | InMotion2  (end-effector) | Distal | Unilaterally | 300 | 5 | 300 | 5 | 12w | AMAT, FMA |
| Prange, 2015.[^21^](#_ENREF_21) | 7 | 53/15 | 7.3/6.8w | 60.3/58 | add-on | ArmeoBoom  (end-effector) | Proximal | Unilaterally | 30 | 3 | 30 | 3 | 6w | SULCS, FMA, Reach distance |
| Rabadi, 2008.[^22^](#_ENREF_22) | 6 | 27/3 | 19/22.5/  22.0d | 79.5/67.8/69.2 | add-on | MIT-MANUS  (end-effector) | Proximal | Unilaterally | 40 | 12 in total | 40 | 12 in total | 2.5w | FMA, FIM, MPS, ARAT, MAS, MSS, pain |
| Reinkensmeyer, 2012.[^23^](#_ENREF_23) | 6 | 13/8/6(unknown) | 65/67mo | 60/61 | alone | Pneu- WREX  (exoskeleton) | Proximal | Unilaterally | 60 | 3 | 60 | 3 | 8w | FMA, BBT, RLAFT-UE, MAL, grip strength |
| Rodgers, 2019.[^24^](#_ENREF_24) | 8 | 613/157 | 233/258  /242d | 59.9/59.4/62.5 | alone | MIT-MANUS  (end-effector) | Proximal | Unilaterally | 45 | 3 | 45 | 3 | 12w | ARAT, FMA, BI, SIS,EQ-5D, pain |
| Takahashi, 2016.[^25^](#_ENREF_25) | 5 | Both | 47.8/46.9mo | 65.2/64.6 | add-on | ReoGo  (end-effector) | Proximal | Unilaterally | 40 | 7 | 40 | 7 | 6w | Brunnstrom stage, FMA, MI, MAS, WMFT, ROM, MAL, FIM, VAS |
| Taveggia, 2016.[^26^](#_ENREF_26) | 7 | Both | 0.5-12mo | 73/68 | add-on | ARMEO Spring  (exoskeleton) | Proximal | Unilaterally | 30 | 5 | 30 | 5 | 6w | FIM, MI, MAS, VAS |
| Timmermans, 2014.[^27^](#_ENREF_27) | 8 | Both | 2.8/3.7y | 61.8/56.8 | alone | Haptic Master  (end-effector) | Both | Unilaterally | 30*2 | 4 | 30*2 | 4 | 8w | FMA, ARAT, MAL, EuroQol-5D, SF-36, activity monitoring |
| Tomic, 2017.[^28^](#_ENREF_28) | 7 | 23/3 | 35.3/37.3d | 56.5/58.3 | add-on | ArmAssist  (end-effector) | Proximal | Unilaterally | 30 | 5 | 30 | 5 | 3w | FMA, WMFT, BI |
| Vanoglio, 2016.[^29^](#_ENREF_29) | 7 | 19/11 | 15.2/17.8d | 72/73 | add-on | glove Gloreha Professional  (exoskeleton) | Distal | Unilaterally | 40 | 5 | 40 | 5 | 6w | MI, 9-HPT, grip strength, pinch strength, QuickDASH |
| Villafane, 2017.[^30^](#_ENREF_30) | 7 | 24/8 | 0.5-12mo | 67/70 | add-on | glove Gloreha Professional  (exoskeleton) | Distal | Unilaterally | 30 | 3 | 30 | 3 | 3w | NIHSS, MAS, BI, MI, QuickDASH, VAS |
| Volpe, 2008.[^31^](#_ENREF_31) | 6 | 20/1 | 35/40mo | 62 / 60 | alone | InMotion2  (end-effector) | Proximal | Unilaterally | 60 | 3 | 60 | 3 | 6w | FMA, MAS, ARAT, SIS, Joint stability, pain, BDS |
| Wolf, 2015.[^32^](#_ENREF_32) | 7 | Both | 115.5/127.1d | 59.1/54.7 | add-on | Hand Mentor Pro  (exoskeleton) | Distal | Unilaterally | 120 | 5 | 120 | 5 | 8w | ARAT, WMFT, FMA |
| Wu, 2012.[^33^](#_ENREF_33) | 7 | Both | 18.00/17.57mo | 55.13/51.30 | add-on | Bi-Manu-Track  (end-effector) | Proximal | Bilaterally | 90 - 105 | 5 | 90 - 105 | 5 | 4w | FMA, MAL, SIS, kinematic variables |
| Yoo, 2013.[^34^](#_ENREF_34) | 5 | 15/7 | 45.8/41.5mo | 50.9/49.7 | add-on | ReoGo  (end-effector) | Proximal | Unilaterally | 30 | 3 | 30 | 3 | 6w | WMFT, BBT, grip strength, MBI |
| Stein, 2018.^35^ | 4 | Both | 66mo | 54.57/58.14 | alone | Amadeo  (end-effector) | Distal | Unilaterally | 60 | 3 | 60 | 3 | 8w | FMA, ROM, strength, MAL, BI,NHPT, MAS |

FMA-UE: Fugl-Meyer Assessment of the Upper Extremity. ARAT: Action Research Arm Test. WMFT: Wolf Motor Function Test. BBT: Box and Blocks Test. 9-HPT: Nine Hole Peg Test. CAHAI: Chedoke Arm and Hand Activity Inventory. AMAT: Arm Motor Ability Test. FIM: Functional Independence Measure. BI: Barthel Index. MAL: Motor Activity Log. mRS: modified Rankin Scale. SIS: Stroke Impact Scale. SF-36: Medical Outcomes Study Short Form 36. QuickDASH: A shorten version of The Disabilities of the Arm, Shoulder and Hand Outcome Measure (DASH). RLAFT-UE: Rancho Los Amigos Functional Test for the Hemiparetic Upper Extremity. EEG: electroencephalogram. MEP: motion evoked potential. sEMG:surface electrocardiograph. VAS: visual analogue scale. ROM: range of motion. SULCS: Stroke Upper Limb Capacity Scale.

**Ⅵ.References of the included studies**

1. Burgar CG, Lum PS, Scremin AME, Garber SL, Van der Loos HFM, Kenney D, et al. Robot-assisted upper-limb therapy in acute rehabilitation setting following stroke: Department of veterans affairs multisite clinical trial. *Journal of rehabilitation research and development*. 2011;48:445-458

2. Bustamante Valles K, Montes S, Madrigal Mde J, Burciaga A, Martinez ME, Johnson MJ. Technology-assisted stroke rehabilitation in mexico: A pilot randomized trial comparing traditional therapy to circuit training in a robot/technology-assisted therapy gym. *Journal of neuroengineering and rehabilitation*. 2016;13:83

3. Calabro RS, Accorinti M, Porcari B, Carioti L, Ciatto L, Billeri L, et al. Does hand robotic rehabilitation improve motor function by rebalancing interhemispheric connectivity after chronic stroke? Encouraging data from a randomised-clinical-trial. *Clinical neurophysiology : official journal of the International Federation of Clinical Neurophysiology*. 2019;130:767-780

4. Conroy SS, Whitall J, Dipietro L, Jones-Lush LM, Zhan M, Finley MA, et al. Effect of gravity on robot-assisted motor training after chronic stroke: A randomized trial. *Archives of physical medicine and rehabilitation*. 2011;92:1754-1761

5. Dehem S, Gilliaux M, Stoquart G, Detrembleur C, Jacquemin G, Palumbo S, et al. Effectiveness of upper-limb robotic-assisted therapy in the early rehabilitation phase after stroke: A single-blind, randomised, controlled trial. *Annals of physical and rehabilitation medicine*. 2019;62:313-320

6. Grigoras AV, Irimia DC, Poboroniuc MS, Popescu CD. Testing of a hybrid fes-robot assisted hand motor training program in sub-acute stroke survivors. *Advances in Electrical and Computer Engineering*. 2016;16:89-94

7. Hesse S, Hess A, Werner CC, Kabbert N, Buschfort R. Effect on arm function and cost of robot-assisted group therapy in subacute patients with stroke and a moderately to severely affected arm: A randomized controlled trial. *Clinical rehabilitation*. 2014;28:637-647

8. Housman SJ, Scott KM, Reinkensmeyer DJ. A randomized controlled trial of gravity-supported, computer-enhanced arm exercise for individuals with severe hemiparesis. *Neurorehabil Neural Repair*. 2009;23:505-514

9. Hsieh YW, Lin KC, Horng YS, Wu CY, Wu TC, Ku FL. Sequential combination of robot-assisted therapy and constraint-induced therapy in stroke rehabilitation: A randomized controlled trial. *Journal of neurology*. 2014;261:1037-1045

10. Hsu HY, Chiu HY, Kuan TS, Tsai CL, Su FC, Kuo LC. Robotic-assisted therapy with bilateral practice improves task and motor performance in the upper extremities of chronic stroke patients: A randomised controlled trial. *Australian occupational therapy journal*. 2019

11. Hung CS, Hsieh YW, Wu CY, Lin KC, Lin JC, Yeh LM, et al. Comparative assessment of two robot-assisted therapies for the upper extremity in people with chronic stroke. *The American journal of occupational therapy : official publication of the American Occupational Therapy Association*. 2019;73:7301205010p7301205011-7301205010p7301205019

12. Klamroth-Marganska V, Blanco J, Campen K, Curt A, Dietz V, Ettlin T, et al. Three-dimensional, task-specific robot therapy of the arm after stroke: A multicentre, parallel-group randomised trial. *The Lancet. Neurology*. 2014;13:159-166

13. Kutner NG, Zhang R, Butler AJ, Wolf SL, Alberts JL. Quality-of-life change associated with robotic-assisted therapy to improve hand motor function in patients with subacute stroke: A randomized clinical trial. *Physical therapy*. 2010;90:493-504

14. Lee MJ, Lee JH, Lee SM. Effects of robot-assisted therapy on upper extremity function and activities of daily living in hemiplegic patients: A single-blinded, randomized, controlled trial. *Technology and health care : official journal of the European Society for Engineering and Medicine*. 2018;26:659-666

15. Liao WW, Wu CY, Hsieh YW, Lin KC, Chang WY. Effects of robot-assisted upper limb rehabilitation on daily function and real-world arm activity in patients with chronic stroke: A randomized controlled trial. *Clinical rehabilitation*. 2012;26:111-120

16. Linder SM, Rosenfeldt AB, Bay RC, Sahu K, Wolf SL, Alberts JL. Improving quality of life and depression after stroke through telerehabilitation. *The American journal of occupational therapy : official publication of the American Occupational Therapy Association*. 2015;69:6902290020p6902290021-6902290010

17. Lo AC, Guarino PD, Richards LG, Haselkorn JK, Wittenberg GF, Federman DG, et al. Robot-assisted therapy for long-term upper-limb impairment after stroke. *The New England journal of medicine*. 2010;362:1772-1783

18. Lum PS, Burgar CG, Shor PC, Majmundar M, Van der Loos M. Robot-assisted movement training compared with conventional therapy techniques for the rehabilitation of upper-limb motor function after stroke. *Archives of physical medicine and rehabilitation*. 2002;83:952-959

19. Masiero S, Armani M, Ferlini G, Rosati G, Rossi A. Randomized trial of a robotic assistive device for the upper extremity during early inpatient stroke rehabilitation. *Neurorehabil Neural Repair*. 2014;28:377-386

20. McCabe J, Monkiewicz M, Holcomb J, Pundik S, Daly JJ. Comparison of robotics, functional electrical stimulation, and motor learning methods for treatment of persistent upper extremity dysfunction after stroke: A randomized controlled trial. *Archives of physical medicine and rehabilitation*. 2015;96:981-990

21. Prange GB, Kottink AI, Buurke JH, Eckhardt MM, van Keulen-Rouweler BJ, Ribbers GM, et al. The effect of arm support combined with rehabilitation games on upper-extremity function in subacute stroke: A randomized controlled trial. *Neurorehabil Neural Repair*. 2015;29:174-182

22. Rabadi MH, Galgano M, Lynch D, Akerman M, Lesser M, Volpe BT. A pilot study of activity-based therapy in the arm motor recovery post stroke: A randomized controlled trial. *Clinical rehabilitation*. 2008;22:1071-1082

23. Reinkensmeyer DJ, Wolbrecht ET, Chan V, Chou C, Cramer SC, Bobrow JE. Comparison of three-dimensional, assist-as-needed robotic arm/hand movement training provided with pneu-wrex to conventional tabletop therapy after chronic stroke. *Am J Phys Med Rehabil*. 2012;91:S232-241

24. Rodgers H, Bosomworth H, Krebs HI, van Wijck F, Howel D, Wilson N, et al. Robot assisted training for the upper limb after stroke (ratuls): A multicentre randomised controlled trial. *The Lancet*. 2019

25. Takahashi K, Domen K, Sakamoto T, Toshima M, Otaka Y, Seto M, et al. Efficacy of upper extremity robotic therapy in subacute poststroke hemiplegia: An exploratory randomized trial. *Stroke*. 2016;47:1385-1388

26. Taveggia G, Borboni A, Salvi L, Mule C, Fogliaresi S, Villafane JH, et al. Efficacy of robot-assisted rehabilitation for the functional recovery of the upper limb in post-stroke patients: A randomized controlled study. *European journal of physical and rehabilitation medicine*. 2016;52:767-773

27. Timmermans AA LR, Monfrance M, Geers RP, Bakx W, Smeets RJ, Seelen HA. . Effects of task-oriented robot training on arm function, activity, and quality of life in chronic stroke patients: A randomized controlled trial. *J Neuroeng Rehabil.* 2014;11

28. Tomic TJ, Savic AM, Vidakovic AS, Rodic SZ, Isakovic MS, Rodriguez-de-Pablo C, et al. Armassist robotic system versus matched conventional therapy for poststroke upper limb rehabilitation: A randomized clinical trial. 2017;2017:7659893

29. Vanoglio F, Bernocchi P, Mule C, Garofali F, Mora C, Taveggia G, et al. Feasibility and efficacy of a robotic device for hand rehabilitation in hemiplegic stroke patients: A randomized pilot controlled study. *BioMed research international*. 2017;31:351-360

30. Villafane JH, Taveggia G, Galeri S, Bissolotti L, Mulle C, Imperio G, et al. Efficacy of short-term robot-assisted rehabilitation in patients with hand paralysis after stroke: A randomized clinical trial. *Hand*. 2018;13:95-102

31. Volpe BT, Lynch D, Rykman-Berland A, Ferraro M, Galgano M, Hogan N, et al. Intensive sensorimotor arm training mediated by therapist or robot improves hemiparesis in patients with chronic stroke. *Neurorehabil Neural Repair*. 2008;22:305-310

32. Wolf SL, Sahu K, Bay RC, Buchanan S, Reiss A, Linder S, et al. The haapi (home arm assistance progression initiative) trial: A novel robotics delivery approach in stroke rehabilitation. *Neurorehabil Neural Repair*. 2015;29:958-968

33. Wu C-y, Yang C-l, Chuang L-l, Lin K-c, Chen H-c, Chen M-d, et al. Effect of therapist-based versus robot-assisted bilateral arm training on motor control, functional performance, and quality of life after chronic stroke: A clinical trial. *Physical therapy*. 2012;92:1006-1016

34. Yoo DH, Cha YJ, Kim SK, Lee JS. Effect of three-dimensional robot-assisted therapy on upper limb function of patients with stroke. *Journal of physical therapy science*. 2013;25:407-409

35. Stein J. Robot-assisted hand training (Amadeo) compared with conventional physiotherapy techniques in chronic ischemic stroke patients: A pilot study. NCT03020576.https://www.clinicaltrials.gov/ct2/show/NCT03020576?id=NCT03020576&draw=2&rank=1. 2018
